# Isolated Intramedullary Lumbar Spine Neurocysticercosis: A Rare Occurrence and Review of Literature

**DOI:** 10.1055/s-0041-1739118

**Published:** 2021-12-15

**Authors:** Anil Dhar, Sanjeev Dua, Hershdeep Singh

**Affiliations:** 1 Department of Neurosurgery, Max Super Speciality Hospital, New Delhi, India

**Keywords:** neurocysticercosis, lumbar, spine, intramedullary, rare

## Abstract

Neurocysticercosis (NCC) is the most common parasitic infection of the central nervous system. Spinal cysticercosis is a rather rare clinical occurrence. Intramedullary (IM) spinal NCC is rarer still. Furthermore, cases of IM-NCC at lumbar levels are few and far between. We present a case of a 35-year-old male patient who was diagnosed to have IM-NCC at L2-3 level and was managed surgically with no recurrence at 2 years of follow-up. A systematic literature review (1992–2020) highlights it to be only the third case reported with exclusive lumbar involvement

## Introduction

Neurocysticercosis (NCC) is the most common parasitic infection of the central nervous system (CNS), caused by the larval form of Taenia solium. 1 Pigs are the intermediate hosts, and humans are the definitive/intermediate hosts. Common risk factors are poor personal hygiene and unsanitary pig raising practices. Spinal cysticercosis is a rather rare clinical occurrence to come across with, as per current literature. 2 With an incidence of 0.7 to 3%, 3 it is common to find it in the combination of intracranial cysticercosis. Spinal NCC can be divided in extradural, intradural subarachnoid (leptomeningeal), and intramedullary (IM) (parenchymal) forms. More common are cases with the dorsal spine involvement than the other parts of the spinal cord. 4 IM presentation is usually the rarest. Here, we present a case of 35-year-old male patient who was diagnosed to have L2-L3 spinal IM-NCC and managed surgically without recurrence at 2 years of follow-up.

## Case Report

A 35-year-old man presented with the complaints of low back ache for 12 years, radiating to right leg for 4 months and numbness extending to lateral side of the sole of right foot. On examination, there was a 30% sensory loss in right S1 dermatome as compared with contralateral limb, with no bladder bowel involvement. Patient had no motor deficit. Magnetic resonance imaging (MRI) of the lumbosacral spine was suggestive of IM cystic lesion at L2-3 hypointense on T1-weighted images and hyperintense on T2-weighted images. MRI brain did not reveal any abnormality. Lumbar puncture and serologic studies were not performed.

With the differential diagnosis of neoplastic lesion, the patient was taken up for posterior laminectomy. L2-3 laminectomy was done. A dural bulge was identified. On durotomy, the cord was found to be enlarged. Under microscopic guidance, posterior longitudinal myelotomy was done, the cysts were approached, and subtotal resection of cysts was done. Intraoperatively, three grayish white cysts were identified. Cysts were found to be adherent to the nerve roots causing their inflammation. As a result, one of the cysts could not be excised and was only decompressed. The remaining two cysts were completely excised. Histopathology revealed it to be NCC.

The patient improved postoperatively. Back pain was relieved, and there was significant reduction in radiating pain. He was started on albendazole (15 mg/kg body weight) for 4 weeks and steroids for 2 weeks. The patient was discharged on the 4th post-operative day. He was followed-up biweekly for the first month. Thereafter, monthly follow-up was done for the next 2 months. MRI done at 6 months confirmed resolution of the cystic lesion. Thereafter, 6 monthly follow-up was done. Patient is symptom free and not on any medication at 2 years of follow-up.

## Discussion

Spinal NCC was originally reported by Rockitansky. 4 Its infrequent incidence (0.7–5.85%), 5 is attributed to the sieve effect provided by subarachnoid layer which filters cysticerci, thus preventing them to pass. IM involvement occurs in less than one-fifth of the cases with intradural pathology. 6 The cysticerci migrate via hematogenous and ventriculoependymal pathways, thus afflicting mainly the dorsal segment of spinal cord primarily as a consequence of high-blood flow. 5

In 2017, the Infectious Diseases Society of America (IDSA) and American Society of Tropical Medicine and Hygiene (ASTMH) 7 recommended clinical practice guidelines for the diagnosis and treatment of NCC. The said guidelines strongly advice prescription of corticosteroids in cases of spinal NCC with spinal cord dysfunction and also as an adjunct to antiparasitic treatment. As evidence to recommend one modality of treatment (medical or surgical) over the other is lacking, authors suggested treatment be planned on case basis and surgical expertise available.

## Review of Literature and Results

The authors searched the PubMed database using keywords “Spinal neurocysticercosis” and “spinal cord neurocysticercosis.” A total of 213 results were obtained which included articles pertaining to both IM and extramedullary (EM) spinal cord NCC lesions.

**Fig. 1:**
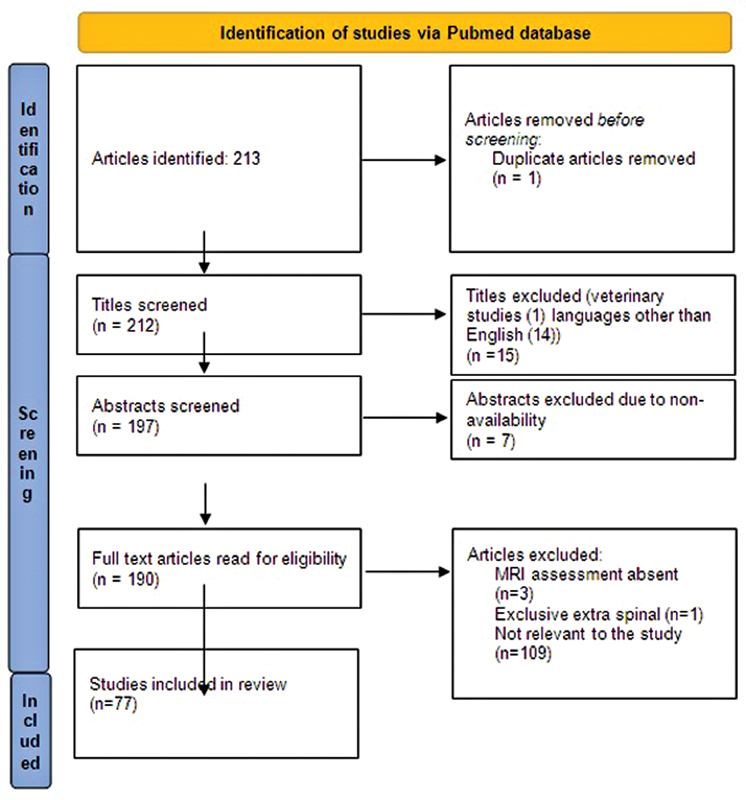
Preferred Reporting Items for Systematic Reviews and Meta-Analyses (PRISMA) flowchart for article selection process.

Research papers reporting exclusive extra spinal involvement, no MRI assessment, published in non-English vernacular, and conducted in nonhuman subjects were excluded from this review ( Fig. 1 ). In the final analysis, 77 articles were shortlisted, encompassing both EM and IM involvement of spinal cord NCC ( Table 1 ). The cumulative number of cases was 147. These include 100 (EM), 46 (IM), and 1 (EM + IM). 8

**Table 1:** Review of current literature

S.no	Authors	Country	Year	Age (yr)	Gender	Compartment	S.C level	Symptoms	Investigations	Mx	Remarks
1	Barrie et al 27	USA	2020	44	F	IM	C5-6	M	M	S	Postop albendazole, steroid
2	Jobanputra et al 28	USA	2020	44	F	IM	C5-C7	P, S	M	S	
3	Garg et al 19	India	2020	51	F	EM	L3-5	P, S	M	C	Malignant (progresses from spine to cranium)
4	Torres-Corzo et al 29	Mexico	2019	39	M	EM	L4-S1	P, M	M	S (endoscopic)	P.O albendazole, Cranial +
				32	F	EM	L5-S1	P, S	M	S (endoscopic)	P.O albendazole, Cranial +
				22	F	EM	L1-4	P, M	M	S (endoscopic)	P.O albendazole, Cranial +
5	Lopez et al 30	Ecuador	2019	58	M	EM	D4-6, D9-11	M, S, B	M	S	Multiple lesions
6	Li et al 31	China	2019		F	EM	CMJ	H	M, IgG antibodies	S	HCP+
7	Shashidha 32	India	2018	55	F	EM	L2-3	H, P	M, E, Eo	C	
8	Almeid 33	Brazil	2018	10	M	IM	D 3-4	M	M	S	P.O albendazole
9	Mast 2	India	2018	30	M	IM	D11	P, M	M, E, CSF antibody+	M	
10	Zhang et al 34	China	2017	59	F	EM	L1-S1	P, B	M, E	S	Postop albendazole given
11	Datta et al 26	India	2017	70	M	IM	D9-10	P, M, B	M	M	P.O albendazole, Operated twice
				23	M	IM	D10-11	P, M, B	M	S	
				24	M	IM	D5-6	P, S	M	O	Refused Rx
12	Yacoub et al 8	USA	2017	49	M	EM	C4-D4, D6-D9	M, S	M	S	Cranial + P. O Albendazole
13	Muralidharan et al 3	India	2017	56	M	EM	CMJ-C4	M, S, B	M, EITB	S	P.O albendazole
14	Pal et al 35	India	2017	44	M	EM	D12-L3, D1-D9	P, M, S	M	S	Operated twice, P. O Albendazole given, mimics arachnoid cyst
15	Yadav et al 36	India	2017	8	M	IM	C5–6	P, M	M	M	
16	Sharma 37	India	2017	48	F	EM	L2-S2	P	M, Eo	S	P.O albendazole
17	Bansal et al 6	India	2017	40	M	EM	L5-S1	P	M	S	P.O albendazole
18	Ranja 25	India	2017	6	M	IM	C4-6	P	M, S. ELISA +	M	
19	Hansberry et al 38	USA	2016	49	M	EM	Post fossa to C2	M, S	M, WBA	S	Cranial +
20	Pant et al 39	India	2016	60	M	IM	D11	M, R, B	M	S	P.O albendazole
				25	F	EM	D12-L2	P, M	M	S	P.O albendazole
21	Torous et al 40	USA	2016	40	M	EM	L4-S1	P, M, S, B	M	S	
22	Valsangkar 41	India	2015	40	M	IM	D10-12	P, M, B	M	S	P.O albendazole
23	Salazar Noguera 42	Guatemala	2015	43	M	IM	C7-D1	M, S	M	S	
24	Hackius 43	Switzerland	2015	46	F	EM	C1-C2, L4–5	H, N, D	M, Eo, E	M	
25	Cárdenas 9	Mexico, India	2015	64	M	EM	CMJ	M		S	30 year study,19 Mexican,8 Indian
				57	M	EM	CMJ	M		C	
				60	F	EM	D1-D7	M, B		C	
				68	M	EM	D12-L3	M, B, R		S	
				55	F	EM	C3-4	M, B		O	
				26	F	EM	C7-D2	M, B, R		C	
				57	F	EM	D5-7	S		C	
				21	M	EM	D5-7	S		C	
				50	M	EM	L4-5	M, B, R		C	
				48	M	EM	L3-4	M		C	VP diversion
				29	F	EM	D4-8	M, B, R		S	
				52	M	EM	L3-5	S, B		S	
				45	F	EM	C1-5, C5-D8	M, B, R		C	
				64	F	EM	D1-2	S		S	
				32	M	EM	C2-3	S, Z		M	
				38	F	EM	L2-4	M		M	
				49	F	EM	CMC-C2	M		C	
				33	F	EM	C3-4, L2-4	S		S	
				62	F	EM	D5-8	M		M	
				50	F	EM	L4	M		C	
				16	F	IM	D11	M		S	
				35	F	EM	D12-L1	M		C	
				45	M	IM	CMJ	M		S	
				NA	NA	IM	D2	M		S	
				16	M	IM	L1	M, B		S	
				39	M	IM	D12	M		S	
				28	M	IM	D1–2	M		S	
26	Chaurasia et al 21	India	2015		M	IM	D11	M, B, P	M, E (CSF, serum)	M	Brown–Sequard Syndrome
27	Wang et al 44	USA	2015	45	M	EM	CMJ	H, P	M, serum antibody +	S	P.O albendazole
28	Ganesan 45	India	2015	32	M	EM	L2-S1	P, B, S	M	S	
29	Han et al 46	South Korea	2014	59	M	EM	L1-5	P, S, M	M	S	Multiple, P.O Albendazole
30	Vecchio et al 47	Italy	2014	23	M	EM	L3-4	H, D	M, E	M	Cranial +
31	Amelot et al 48	France	2014	48	M	EM	CVJ	M, H, PS	M	C (VP shunt)	Case series of 3 cases, 2 had spinal involvement
				25	F	EM	L4-S2	H, P, AMS	M	EVD, S	
32	Kim et al 49	South Korea	2014	64	M	EM	D12-L1, L3-4	H, M, B	M	S (VP shunt+ laminectomy)	Hydrocephalus +, P.O albendazole
33	Qazi 14	India	2014	19	M	IM	D11-L1	M, B	M	S	
34	Yoo et al 50	South Korea	2014	42	M	EM	D11-S1	P	M, S.E	S	P.O albendazole
35	Lacoangeli 51	Italy	2013	44	F	EM	L4-5	P, M, S, B	M	S	P.O albendazole
36	Chandramohan 15	India	2013	15	M	IM	L1	M, B, R	M, Western blot	M	
37	Rice et al 22	USA	2012	42	M	IM	D10-D11	M, S	M	S	Brown–Sequard Syndrome
38	De Deo et al 52	Italy	2012	49	M	EM	D6-8, D10-11	H, M	M	S	Cranial + , P.O Albendazole
39	Callacondo et al 53	Peru	2012	NA	NA	All 18 EM	LS M.C				Out of 55 patients with cranial NCC (intraparenchymal + basal cisterns) 18 pt had spinal involvement all EM
40	Jain et al 16	India	2012	20	M	IM	C2	Z, M, B	M, IgG	M	Lost to follow up, Cranial+
41	Shin et al 54	South Korea	2012	48	M	EM	D12-S1	M, B	M	S	P.O albendazole
42	Agale 10	India	2012	38	M	IM	D10-11	M	M	S	P.O albendazole
43	Kapu et al 55	India	2012	38	F	EM	D12-L1	P, M, S	M	S	P.O albendazole, Cysticercal abscess
44	Bin Qi et al 56	China	2011	40	F	IM	D4-5	M, B, R		S	P.O anticyststicercal agents
45	Seo et al 57	South Korea	2011	59	M	EM	D12-L1, L4-5	H, V	M, Eo	S	P.O albendazole, ocular symptoms, Visual defects persisted
46	Jongwutiwes 58	USA	2011	59	F	EM	L1–4	M, S, B	M, S.E	S	
47	Park 59	South Korea	2011	72	M	EM	L5-S1	P, M	M	S	Cranial + HCP+
48	Lambertucci 60	Brazil	2011	23	M	IM	C3–5	P, M	M	S	P.O albendazole
49	Azfar 61	India	2011	10	F	IM	D2	M, S, B, R	CSF E	M	
50	Vij et al 23	India	2011	20	M	IM	D10-11	P, M, S, B	M	S	Coexisting IM Schwannoma
51	Jang et al 62	South Korea	2010	50	F	EM	L5-S1	P	M	S	Reoperated, P.O Albendazole
52	Boulos et al 63	Canada	2010	35	F	EM	CMJ	H, S	M	S	P.O albendazole, Cranial leptomeningeal enhancement +
53	Lim et al 64	South Korea	2010	42	M	EM	C2-L2	M	M	S (D3–5, L1–3)	H/O HCP +, P.O Albendazole
54	Choi et al 65	South Korea	2010	43	F	EM	L5-S1	P, M, S	M	S	PIVD L5-S1, P.O Albendazole
55	Gonçalves et al 66	USA	2010	62	M	IM	D11	P, M, S, B	M	S	
56	Chibber et al 67	India	2009	38	F	IM	D5-6	P, M, B	M, E	M	
57	Shin 68	South Korea	2009	45	M	EM	C1-L1	M, B	M, CSF ELISA	S	Cranial + HCP +, P.O albendazole
58	Kasliwal et al 69	India	2008	34	M	EM	C1-C2	P, M, H	M	S	P.O. albendazole
59	Izci et al 12	USA	2008	70	M	IM	D11-L1	M, B, R	M	S	P.O albendazole
60	Paterakis 70	Greece	2007	60	M	EM	L3, L5-S1	P, M, B	M	S	P.O Albendazole
61	Ahmad 11	India	2007	8	F	IM	D8	P, M, B	M	S	P.O albendazole
				35	F	IM	D1-2	P, M, S, B	M	S	P.O albendazole
62	Guedes-Corrêa 13	Brazil	2006	53	F	IM	D12-L1	P	M	S	
63	Delobel 71	France	2004	45	M	EM	L3-4	P, M, B	M, E (CSF, serum)	S	HIV + cranial +, Brown Sequard syndrome, P.O Albendazole
64	Torabi et al 17	USA	2004	35	M	IM	C4, D4-9	H, P, M, B	M	M	Cranial + HCP +, Multilevel
65	Alsina et al 18	USA	2002	38	M	EM	L2-3	M, B	M	C	Cranial +
				14	F	EM	C5-D1	M	M	S	
				36	M	EM	C5	M	M	S	Cranial+
				40	M	EM	FM	H	M	C	
				28	F	IM	C1	M	M	M	Cranial +
				80	M	EM	D4-5, D7-9	P	M	S	
66	Colli 72	Brazil	2002	15	F	EM	D9	M	M	S	HCP+
				23	F	EM	D2-L1	P, M	M	S	HCP+
				24	F	EM	L2-5	P, S	M	S	
				36	F	EM	D11-L5	M, B, R	M	O	HCP+
				40	F	EM	C5-6	M, B	M	S	HCP+
				43	F	EM	L3-5	P, M	M	S	HCP+
				46	F	EM	D1-2	M, S	M	S	HCP+
				46	F	EM	D9-L1	P, M, B, R	M	S	HCP+
				22	M	EM	D1	P	M	O	HCP+
				24	M	EM	D8-L2	P	M	S	
				24	M	EM	L3-4 S1	P, S, M	M	S	HCP+
				51	M	EM	C3-7		M	S	
67	Sheehan 73	USA	2002	16	F	IM	C1-2	S	M	S	P.O praziquantel
68	Homans 4	USA	2001	5	F	IM	D11-12	P, B	M, EITB	S	Cranial + operated twice
69	Mathuriya et al 74	India	2001	28	M	IM	D1	P, M, S, B	M	S	
				55	M	IM	D1-2	P, M, S, B	M	S	
				50	M	IM	D11	P, M, S, B	M	S	
70	Gaur et al 24	India	2000	22	F	IM	D8	M, S, B	CSF ELISA	M	
				27	F	IM	D5-6	M, S, B, R	CSF ELISA	M	
71	Ciftçi et al 75	USA	1999	30	F	EM	C2-4	H, P	M	NA	HCP +
72	Garg et al 76	India	1998	11	M	IM	D9	M, S, B	M, CSF, S. ELISA	M	Cranial+
				10	M	IM	D8	M, S, B	M	M	
73	Lau et al 77	Hong Kong	1998	35	M	EM	D11-S1	M, S, HL	M	S	Cranial+
74	Davies 78	Australia	1996	40	M	EM	C3-6	M, H, PS	M	S	Cranial + CSF diversion done multiple times, P.O Praziquantel
75	Corral 20	Spain	1996	20	F	IM	C	Z, S, M	M, E	M	Cranial +
76	Isidro-Llorens 79	Spain	1993	30	F	EM + IM	C7-L2, IM at D	M	M	S	Operated twice, P.O Praziquantal
77	Bandres et al 80	USA	1992	34	M	EM	C2, S1-L3	P	M	M	A case series of 5 patients, ventricular dilation +

Abbreviations: CSF, cerebrospinal fluid; CMJ, cervicomedullary junction; CVJ, craniovertebral junction; EM, extramedullary; EITB, enzyme-linked immunoelectrotransfer blot; ELISA, enzyme-linked immunosorbent assay; IgG, immunoglobulin G; IM, intramedullary; VP, ventriculoperitoneal.

The literature review done by authors revealed only 33 articles (case reports and series) pertaining to the IM-NCC, the earliest being published in 1996.It translates to three articles being published from 1996–2000, followed by nine articles being in print from 2001–2010, and finally 21 articles from 2011–2020. Evidently, there has been increased scientific interest in spinal NCC. Increase in data availability will help to make more evidence-based treatment guidelines possible.

The review brings forth the fact that such cases were found not only in countries of Asia, Mexico but also in countries of the developed world. Eighteen such publications originate from India, followed by eight in the United States, three in Brazil, and one each in China, Guatemala and Spain. A 30-year long (1980–2010) combined research study was undertaken by clinicians from Mexico and India 9 .In most instances, the patients in the developed nations have a history of travel to the endemic region or a history of immigration. 10 11 12 It not only highlights the significance of cultural and environmental impact in this parasitic disease but also the need to consider this rare entity as a differential in such patients by clinicians in the developed world.

The patients with IM-NCC ranged in age from 5 to 70 years with an average of 31.06 years. Among the 46 patients of IM-NCC, 30 were male and 15 were female. In one of the study, this information was not provided. 9

Spinal NCC has been reported to occur most commonly in the dorsal spine. The authors found majority ( *n =* 32) of the IM-NCC has been reported to be located in dorsal or dorsolumbar levels, including two cases occurring at D11-L1 and D12-L1. 13 14 It is followed by cervical and cervicodorsal region ( *n =* 12). Highly sporadic occurrence has been reported in the lumbar region. Two of 46 cases of IM-NCC occur purely in the lumbar region. 9 15 Our patient is only the third reported case with pure lumbar involvement. With such an unusual occurrence, the diagnosis of the NCC was overlooked, and intraoperative findings were contrasting to our preoperative assessment, compelling us to share our experience.

Isolated spinal NCC is not a common occurrence. Concomitant cranial lesions are usually present. Of the 147 cases thus reported, 39 patients were known to have a concurrent or a history of cranial involvement. Six of the patients with IM-NCC had such a finding. 4 16 17 18 19 20

Eighteen of patients with spinal NCC were reported to have hydrocephalus. Only one of these patients had IM-NCC, 17 who also had an evidence of cranial NCC.

The most common symptoms in patients with IM-NCC were those of motor involvement (40), followed by bladder involvement (26), back pain (21), sensory involvement (17), and bowel involvement (6). Two patients had complaints of seizures while headache was seen in one patient. These patients had cervical spine and brain lesions. Two patients (dorsal level IM-NCC) presented with Brown–Séquard syndrome (dorsal lesion). 21 22 One of the patients was found to have a coexisting schwannoma at D10-11 lesion. 23 Patients with pure lumbar involvement had motor symptoms and bladder bowel involvement. 9 15

MRI is the investigation of choice for spinal NCC. Research papers with no MRI assessment were excluded from this review. MRI is the most essential to make diagnosis of spinal pathology, its level, and compartment involved. Other investigations may not always be helpful. In only 12 of the cases of IM-NCC, antibodies were detected by various techniques, including enzyme-linked immunosorbent assay (ELISA), Western blot, and enzyme-linked immunoelectrotransfer blot (EITB). 24 25

In the review, it was concluded that surgery was the main modality of treatment ( *n =* 29), while 16 patients were managed medically with anticysticercal agents (albendazole, praziquantel). One of the patients refused any treatment. Postop medical treatment was given to 12 patients. Redo surgery was required in two cases, both were in dorsal region. 4 26 There is no conclusive evidence pointing to advantage of one modality over the other. Case-based decisions are made, and patients are treated, according to clinical expertise available

## Conclusion

IM spinal NCC is a rare occurrence, even scarcer in the lumbar regions. To the author's knowledge, this case study is only the third to be reported in global data, thus adding up to the current literature.

Given its rarity, it is highly likely that such a diagnosis be ignored at the outset. It is prudent to consider this differential, especially in relation to patient history, travel history, personal history, and cultural background, to avoid any surprise. Advocated by clinical judgement, although medical treatment has been followed by similar results, surgical intervention remains the mainstay of treatment of spinal NCC. Although clinicians do prescribe steroids and antiparasitic agents in postop period, strong evidence-based guidelines are needed, necessitating more high-quality research. Steady follow-up is crucial to detect recurrence.
